# Vascularized Lymph Node Transfer Surgery for Successful Resolution of Long-Standing Lower Limb Lymphedema With Charles Excision: A Case Report

**DOI:** 10.7759/cureus.59000

**Published:** 2024-04-25

**Authors:** Pankhuri Garg, Firoz Borle

**Affiliations:** 1 General Surgery, Jawaharlal Nehru Medical College, Datta Meghe Institute of Higher Education and Research, Wardha, IND

**Keywords:** multidisciplinary approach, color doppler, lympho scintigraphy, surgical management, charles procedure, lymphedema

## Abstract

Lymphedema, a chronic condition characterized by abnormal swelling resulting from impaired lymphatic drainage, poses significant challenges in clinical management, especially when conventional therapies prove ineffective. This case report elucidates the successful resolution of long-standing lower limb lymphedema in a 35-year-old male through innovative surgical interventions. Despite enduring symptoms for 15 years and undergoing various treatments without improvement, the patient achieved remarkable relief following vascularized lymph node transfer surgery combined with Charles excision. This multidisciplinary approach aimed to restore lymphatic function and alleviate tissue bulk, addressing the condition's functional and cosmetic aspects. Preoperative evaluations, including imaging studies confirming grade IV lymphedema, guided surgical planning and contributed to the successful outcome. Postoperatively, despite wound dehiscence, prompt management facilitated satisfactory wound healing, underscoring the importance of meticulous postoperative care. This case underscores the significance of surgical intervention in managing refractory lymphedema and emphasizes the need for tailored treatment strategies to optimize patient outcomes. Further research and clinical experience are warranted to refine surgical techniques and identify optimal patient selection criteria, advancing the management of this challenging condition.

## Introduction

Lower limb lymphedema is a chronic condition characterized by the accumulation of lymphatic fluid, leading to persistent swelling, discomfort, and impaired mobility. It often results from damage to or dysfunction of the lymphatic system, commonly observed following surgical procedures, radiation therapy, trauma, or parasitic infections such as filariasis [1]. Conservative management strategies, including compression therapy, physical therapy, and manual lymphatic drainage, form the cornerstone of treatment for lymphedema. However, these approaches may provide only partial relief and fail to address underlying lymphatic dysfunction. In cases of refractory lymphedema, surgical interventions are increasingly being considered to alleviate symptoms and improve patient outcomes [2].

Vascularized lymph node transfer (VLNT) is a promising surgical technique for managing lymphedema. By transplanting healthy lymph nodes with their associated blood supply into the affected area, VLNT aims to restore lymphatic function and promote drainage of excess fluid [3]. Additionally, Charles excision, involving removing fibrofatty tissue from the affected limb, can complement VLNT by reducing tissue bulk and facilitating lymphatic flow [4]. Despite the growing interest in surgical interventions for lymphedema, more data is needed on the long-term outcomes and efficacy of these procedures. Case reports documenting successful outcomes following VLNT combined with Charles excision provide valuable insights into the potential benefits of surgical management for refractory lymphedema [5]. This case report presents a 35-year-old male with long-standing lower limb lymphedema secondary to filariasis who underwent VLNT with Charles excision for symptom relief. The report highlights the clinical presentation, surgical intervention, postoperative course, and outcomes of this case, underscoring the role of surgical therapy in managing chronic lymphedema.

## Case presentation

We present a case involving a 35-year-old male who sought care at the outpatient department of a tertiary care hospital, presenting with chronic edema in the right leg, persisting for 15 years, accompanied by difficulty walking and multiple episodes of lymphangitis. He also reported a history of itching and increased swelling during specific seasons, with subsequent relief of symptoms. During the medical history assessment, the patient described a gradual worsening of the edema over the past decade. He had no known comorbidities or surgical history. Further information was obtained from the patient's medical records from a local hospital, where various treatments, including allopathic and homeopathic approaches, had been administered.

Upon physical examination, the medical team observed noticeable edema in the right leg impeding proper ambulation. Preoperatively, the patient was advised to elevate the limb and apply a crepe bandage for two months to aid in manual lymphatic drainage. Additionally, lymphoscintigraphy was recommended. The patient underwent conservative management with multi-layer bandaging for two months. Palpation revealed discomfort and pain, leading to the recommendation for surgical intervention.

**Figure 1:**
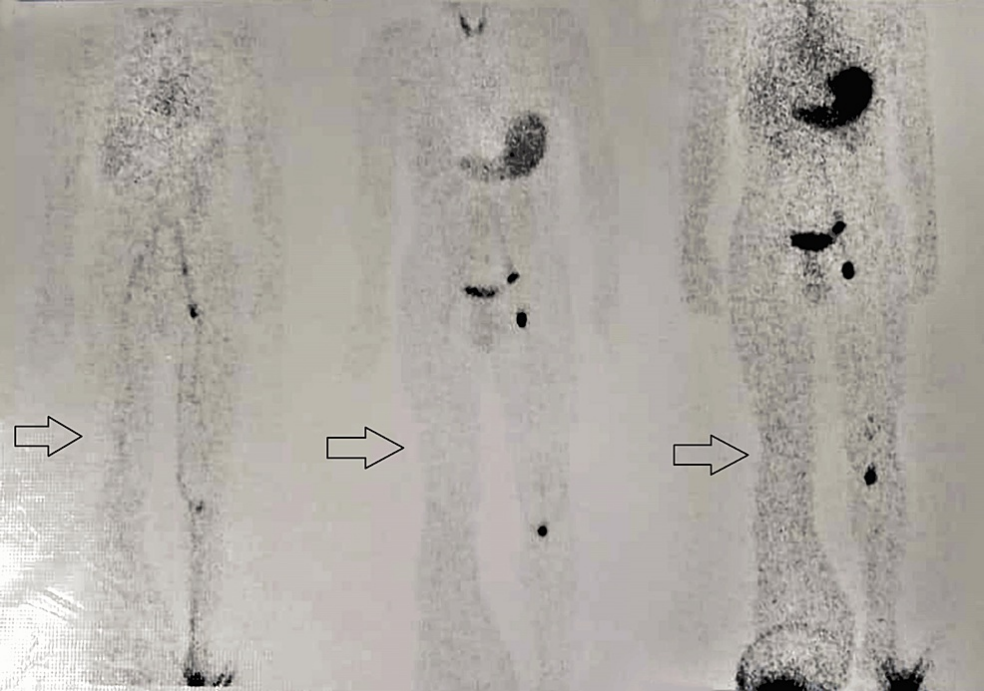
Stasis and dermal back flow in the right lower leg, along with nonvisualization of proximal nodes suggestive of grade IV lymphedema

Upon admission to the surgical inpatient department, analgesics were initiated for symptomatic pain relief. Subsequent blood tests and a right leg X-ray yielded normal results. Due to persistent concerns, a color Doppler study and lymphoscintigraphy were advised. MRI findings revealed diffuse subcutaneous edema and dilated lymphatic channels in the right leg. Lymphoscintigraphy indicated significant tracer stasis and dermal backflow in the right lower leg, suggesting grade IV lymphedema (Figure 1). A color Doppler study showed no significant arterial or venous flow abnormalities, with evidence of well-defined collections in the right leg.

The medical team thoroughly explained the condition and recommended surgery to the patient and their relatives. After obtaining written consent, preoperative preparations were initiated, including antibiotic administration. A preanesthetic evaluation confirmed the patient's suitability for surgery, leading to their transfer to the operating theater under nil per oral (NPO) status. Circumference measurements of both lower limbs were compared.

**Figure 2:**
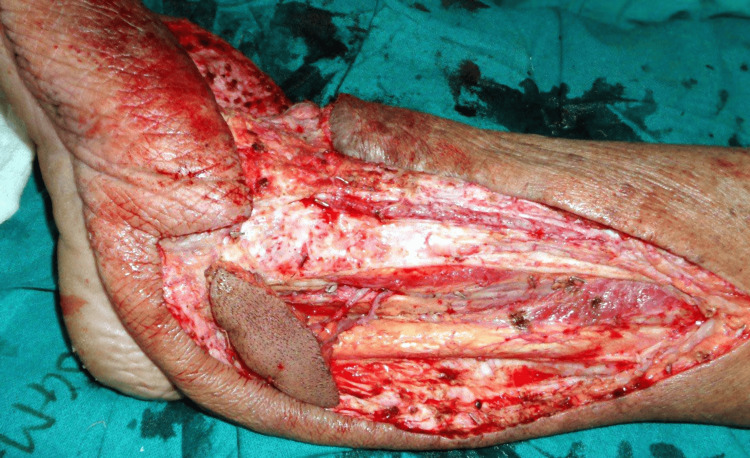
Vascularized submental lymph node transfer for right lower limb lymphedema

Under general anesthesia, the surgical team performed vascularized right submental lymph node transfer for right lower limb filarial lymphedema (Figure 2).

**Figure 3:**
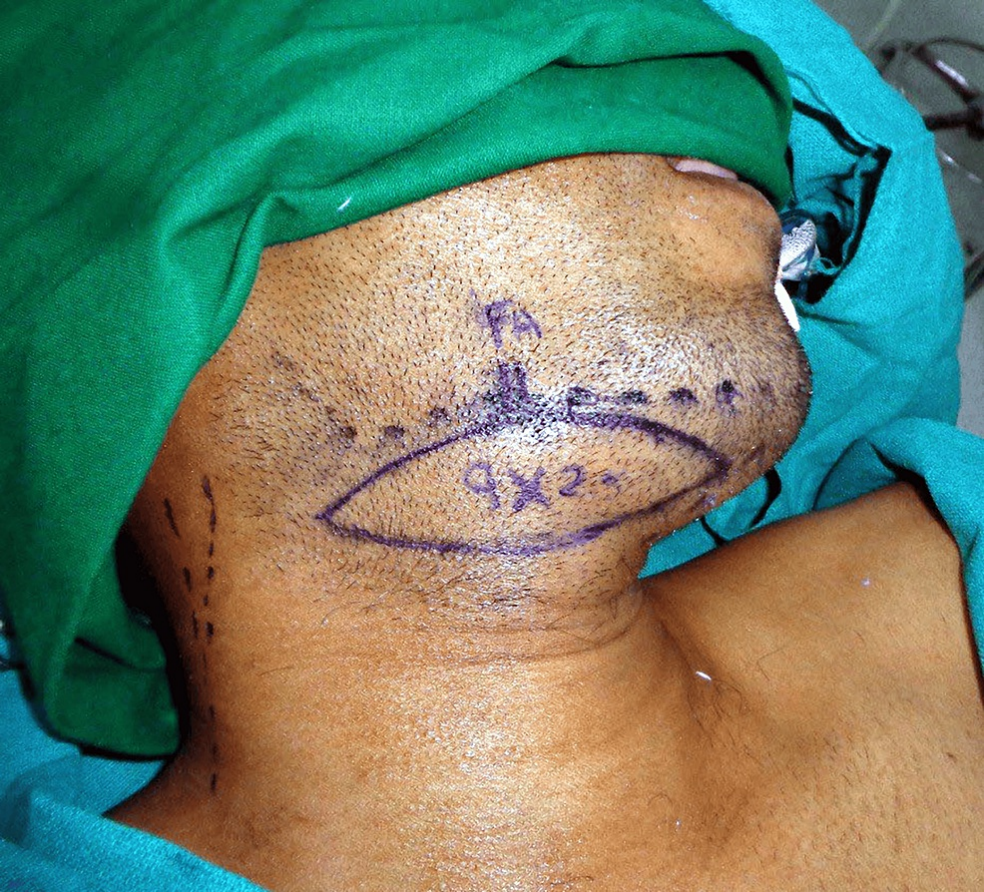
Right submental flap marked extending from the midline to the anterior border of right sternocleidomastoid muscle and lower border of the marked mandible

**Figure 4:**
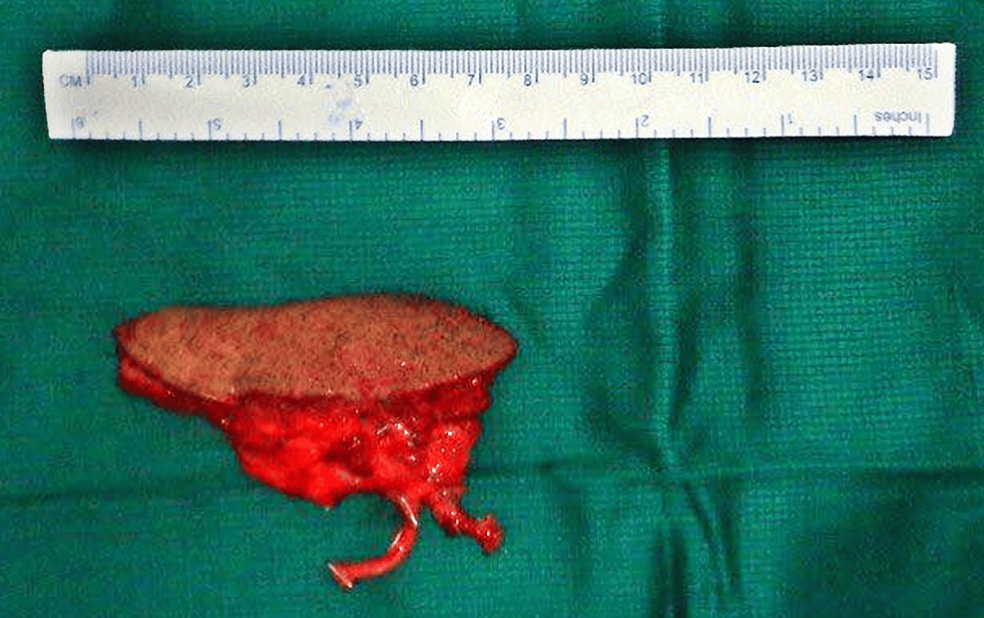
Submental flap along with flap artery and flap vein

The lymphedematous portion of the distal right lower limb was excised, preserving the right great saphenous vein and posterior tibial vessels. A compression dressing was applied, and the tourniquet was deflated. The right submental flap was raised and anastomosed to the right posterior tibial artery and vein (Figures 3, 4). The procedure concluded without complications, and the patient was transferred to the recovery room for observation.

**Figure 5:**
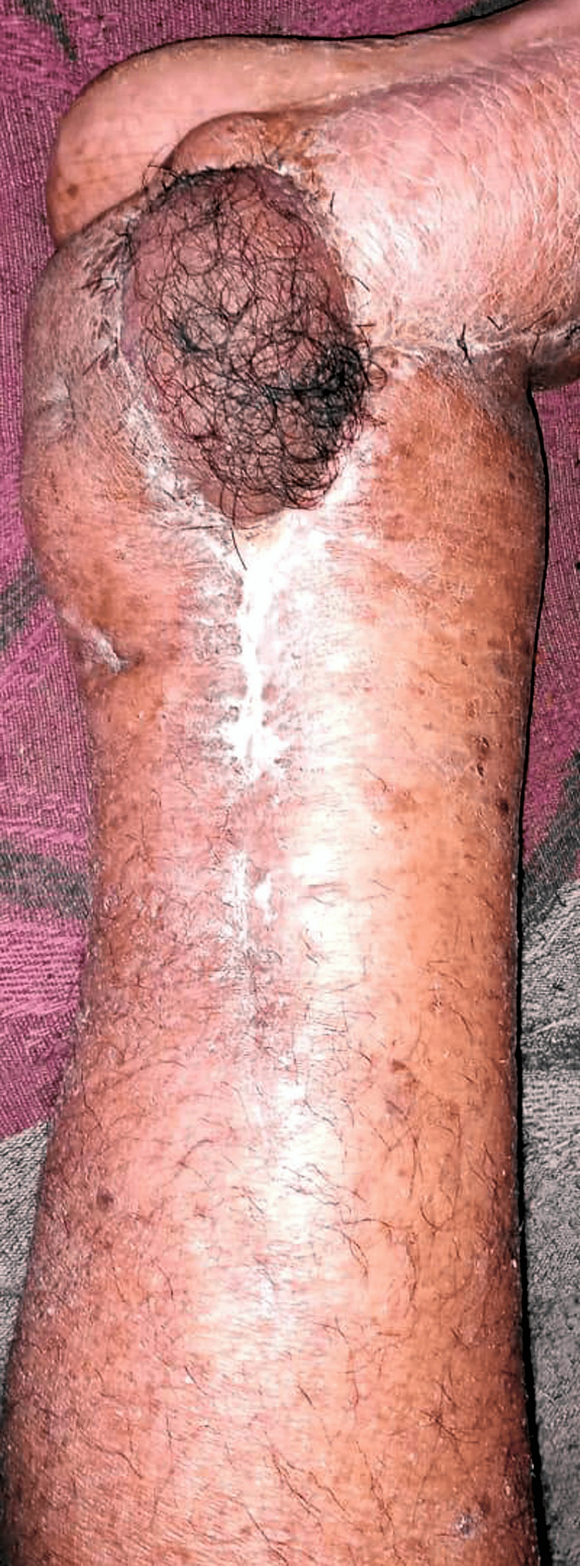
Postoperative image showing healthy flap with a healed suture line

After 24 hours, the patient was transferred to the surgical department. A dressing examination revealed no evidence of bleeding or suture disruption. The bright red color of the flap confirmed successful hemostasis. Postoperatively, the patient received daily dressings, injectable antibiotics, and analgesics. On postoperative day 7, wound dehiscence occurred, necessitating daily dressings with betadine, hydrogen peroxide, and normal saline. Secondary suturing with 1-0 vicryl was performed on postoperative day 11. By postoperative day 30, the wound had healed well, and sutures were removed (Figure 5).

## Discussion

Managing long-standing lower limb lymphedema presents a significant challenge, particularly when conservative measures fail to provide adequate relief. This case report demonstrates the successful resolution of chronic lymphedema using vascularized lymph node transfer surgery combined with Charles excision. Chronic lymphedema, as observed in our patient, often results from lymphatic obstruction or dysfunction, leading to progressive swelling, impaired lymphatic drainage, and recurrent infections [6]. Despite the implementation of conservative measures such as compression therapy and manual lymphatic drainage, many patients with advanced lymphedema experience persistent symptoms and functional limitations [7]. Surgical intervention is necessary to address the underlying pathology and improve patient outcomes.

VLNT has emerged as a promising surgical option for treating lymphedema. By transplanting healthy lymph nodes to the affected area, VLNT aims to restore lymphatic function and facilitate fluid drainage, thereby reducing edema and decreasing the risk of complications [8]. In our case, using a vascularized lymph node flap from the submental region provided a robust source of lymphatic tissue for revascularization in the affected lower limb, and in addition to VLNT, Charles excision played a crucial role in the surgical management of our patient's lymphedema. Charles excision involves the removal of fibrofatty tissue from the affected limb, thereby reducing bulk and improving limb contour [9]. By combining VLNT with Charles excision, our surgical approach addressed both lymphedema's functional and cosmetic aspects, leading to comprehensive symptom relief and enhanced patient satisfaction [10].

Despite the overall success of the surgical intervention, our case was manageable. Postoperative wound dehiscence occurred, necessitating secondary suturing and prolonged wound care. While wound complications are common following complex surgical procedures, they highlight the importance of meticulous postoperative management and patient monitoring. By promptly addressing wound issues and providing appropriate care, we achieved satisfactory wound healing and overall patient recovery. Moving forward, the findings from this case report underscore the potential benefits of integrating VLNT with Charles's excision in the management of refractory lower limb lymphedema. Further research is warranted to evaluate the long-term outcomes and optimal patient selection criteria for these surgical interventions. Additionally, continued advancements in surgical techniques and rehabilitation protocols will improve the efficacy and safety of lymphedema surgery.

## Conclusions

In conclusion, the successful resolution of long-standing lower limb lymphedema in our patient through VLNT surgery combined with Charles excision underscores the efficacy of surgical intervention in managing refractory cases. This comprehensive approach significantly improved symptoms and functional outcomes by addressing the underlying lymphatic dysfunction and excess tissue bulk. Despite postoperative wound complications, meticulous management ensured satisfactory wound healing and overall patient recovery. This case highlights the importance of integrating advanced surgical techniques into the treatment paradigm for lymphedema, offering hope for patients with persistent symptoms resistant to conservative measures. Further research and refinement of surgical protocols will continue to enhance the efficacy and safety of lymphedema surgery, ultimately improving the quality of life for affected individuals.
